# Demographics and treatment of patients with primary membranoproliferative glomerulonephritis in Japan using a national registry of clinical personal records

**DOI:** 10.1007/s10157-023-02387-1

**Published:** 2023-07-29

**Authors:** Naoki Nakagawa, Tomonori Kimura, Ryuichi Sakate, Yoshitaka Isaka, Ichiei Narita

**Affiliations:** 1 Division of Cardiology, Nephrology, Pulmonology and Neurology, Department of Internal Medicine, Asahikawa Medical University, 2-1-1-1 Midorigaoka-Higashi, Asahikawa, Japan; 2 Reverse Translational Research Project, Center for Rare Disease Research, National Institutes of Biomedical Innovation, Health and Nutrition (NIBIOHN), Ibaraki, Japan; 3 Laboratory of Rare Disease Resource Library, Center for Rare Disease Research, National Institutes of Biomedical Innovation, Health and Nutrition (NIBIOHN), Ibaraki, Japan; 4 Department of Nephrology, Osaka University Graduate School of Medicine, Suita, Japan; 5 Division of Clinical Nephrology and Rheumatology, Kidney Research Center, Niigata University Graduate School of Medical and Dental Sciences, Niigata, Japan

**Keywords:** Clinical characteristics, Membranoproliferative glomerulonephritis, Nephrotic syndrome, Registry

## Abstract

**Background:**

Membranoproliferative glomerulonephritis (MPGN) is a rare glomerular injury that causes nephrotic syndrome and end-stage kidney disease. The nationwide demographics and treatment of Japanese patients with primary MPGN have not yet been reported.

**Methods:**

We collected clinical personal records of patients with primary MPGN between 2015 and 2018 from the national registry organized by the Japanese Ministry of Health, Labour, and Welfare and investigated the characteristics of primary MPGN throughout Japan.

**Results:**

Of 258 patients with primary MPGN, 199 and 59 showed nephrotic and non-nephrotic syndrome, respectively. The median age at onset was higher in patients with nephrotic syndrome than in those with non-nephrotic syndrome (45 [24–63] vs. 35 [14–53] years, respectively; P = 0.010). The use of oral prednisolone was significantly higher in patients with nephrotic syndrome than in those with non-nephrotic syndrome (73.9% vs. 59.3%, respectively; P = 0.032). When patients were divided into three age groups: adolescent and young adult group (≤ 39 years; n = 80), middle adult group (40–64 years; n = 111), and older adult group (≥ 65 years; n = 67), the use of oral prednisolone, cyclosporine, and mizoribine was significantly higher in the adolescent and young adult group than in the middle adult group. The mean dosage of oral prednisolone and mizoribine showed no differences among the three age groups.

**Conclusion:**

The national registry of clinical personal records of primary MPGN could provide an informative insight into the characteristics, clinical features, and treatment approaches for patients with primary MPGN in Japan.

**Supplementary Information:**

The online version contains supplementary material available at 10.1007/s10157-023-02387-1.

## Introduction

Primary membranoproliferative glomerulonephritis (MPGN), a rare cause of nephrotic syndrome and end-stage kidney disease (ESKD), is a pattern of injury with characteristic mesangial cellularity and thickening of glomerular capillary walls due to the subendothelial deposition of immune complexes or complement factors [1, 2] with a global incidence of 2 per million population (pmp)/years, in comparison to 6 pmp/year for minimal change disease (MCD), 8 pmp/year for focal segmental glomerulosclerosis (FSGS), and 12 pmp/year for membranous nephropathy (MN) [3]. In Japan, the Japan Renal Biopsy Registry (J-RBR), a nationwide, web-based registry of renal biopsies, reported that the prevalence of MPGN was 2.6%, whereas MCD, FSGS, and MN were 46.2%, 9.8%, and 30.4%, respectively [4, 5]. Furthermore, the Japan Nephrotic Syndrome Cohort Study (JNSCS), which enrolled 374 Japanese patients with primary nephrotic syndrome, reported that the prevalence of MPGN was 2.4%, whereas MCD, FSGS, and MN were 41.3%, 10.0%, and 40.0%, respectively [6], suggesting that primary MPGN is also rare compared to other primary nephrotic syndromes in Japan.

Although several studies on primary MPGN have been conducted in Japan [7–12], most studies are either regional or conducted on a small scale. No cohort study with more than 100 patients has reported the distribution of patients with primary MPGN. Therefore, large-scale demographics, clinical characteristics, and treatment data of Japanese patients with primary MPGN are needed for better healthcare services, to improve treatment, and to provide a reference for comparisons with future studies and medical administration.

Recently, large-scale data, comprising information from various sources, including electronic health records, administrative or claims data, and data from self-monitoring devices, have become a controversial topic in medical and healthcare research [13–15]. Clinical personal records are a Japanese nationwide administrative database of public expenditure for refractory diseases maintained by the Japanese Ministry of Health, Labour, and Welfare to register and certify intractable diseases [16, 17]. This database started collecting primary MPGN data in 2015, which helped investigate the clinical features of patients with primary MPGN in routine practice. This study aimed to describe the demographics and treatment of primary MPGN in Japan using a nationwide registry of clinical personal records.

## Materials and methods

### Overviews of clinical personal records

This cross-sectional study used data from clinical personal records, a nationwide administrative database of public expenditure for refractory diseases (so-called the “National Database of Designated Incurable Diseases of Japan”) including primary MPGN, throughout Japan, maintained by the Japanese Ministry of Health, Labour, and Welfare [18]. The records prospectively and annually collected demographic data (age, sex), chronic kidney disease (CKD) classification based on glomerular filtration rate and albuminuria, medication use (presently used and maximum dosage of present treatment within 6 months), and therapeutic responses (steroid-resistant [never achieved remission]). The data were registered only after being reviewed by certified nephrologists. However, the database does not include survival data, such as death. The results presented in this study are original and different from the statistics produced or published by the Ministry of Health, Labour, and Welfare of Japan.

### Study population

**Fig. 1:**
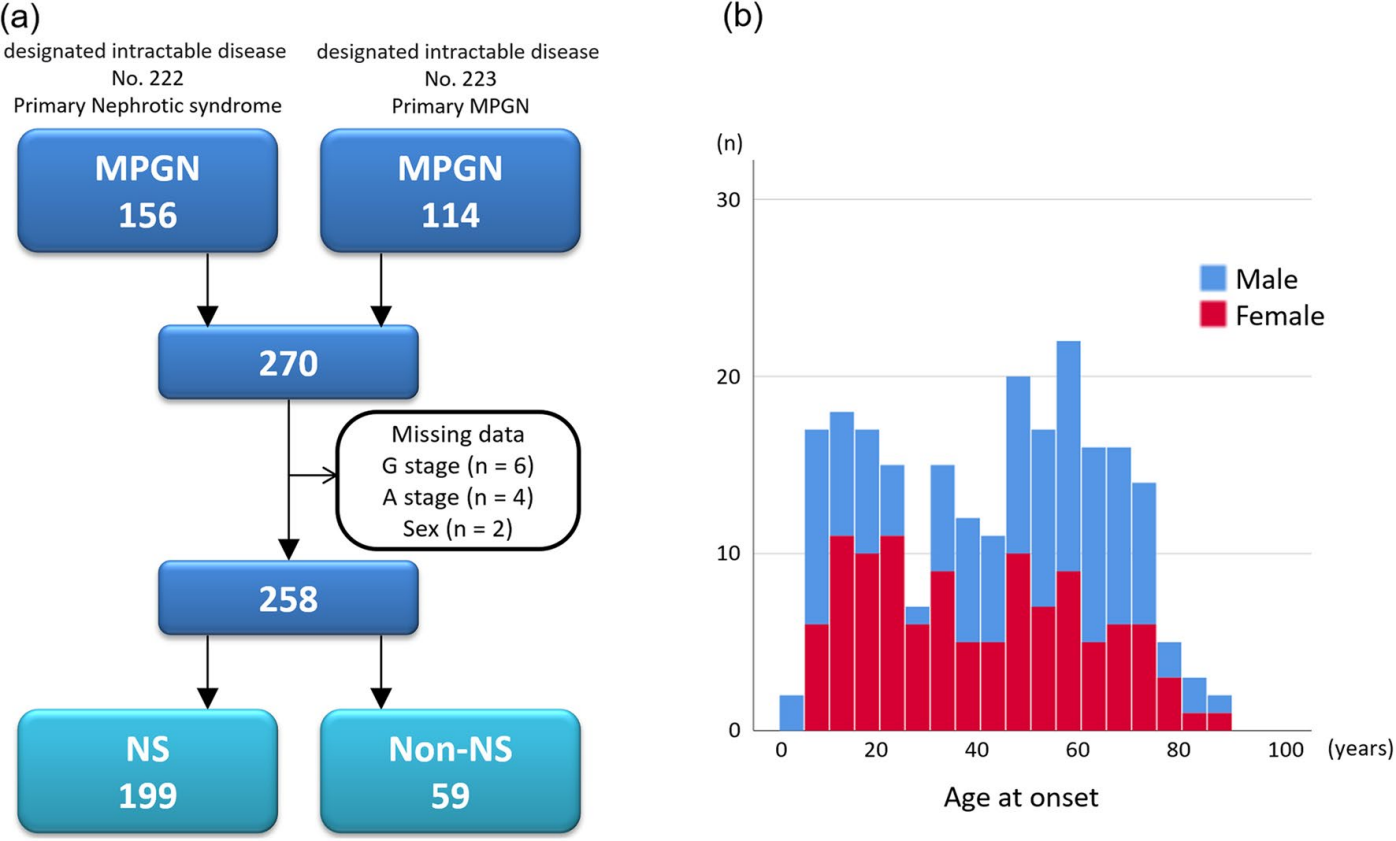
**a** Flow diagram for patient selection from the national registry of clinical personal records of primary membranoproliferative glomerulonephritis (MPGN). **b** Distribution of glomerulopathies in the national registry of primary membranoproliferative glomerulonephritis by sex and age (in years) at onset

The present study used clinical personal records of patients with primary MPGN types I and III from 2015 to 2018. Primary MPGN was diagnosed based on renal biopsy findings in the absence of type II MPGN (dense deposit disease) and any secondary MPGN [11], such as autoimmune diseases (lupus nephritis, IgA vasculitis, and other types of vasculitis), paraproteinemia (amyloidosis, cryoglobulin, heavy chain deposition, and light chain deposition), infectious diseases (streptococcal and staphylococcal infections, hepatitis B and C, human immunodeficiency virus, parvovirus B19, bacterial endocarditis, shunt nephritis), tumors (malignant lymphoma and leukemia), genetic diseases associated with complement dysfunction, or liver diseases (liver cirrhosis and antitrypsin deficiency). Nephrotic syndrome was diagnosed based on massive proteinuria (≥ 3.5 g/day) and hypoalbuminemia (serum albumin ≤ 3.0 g/dL) [19]. In 2015, primary MPGN was approved as a designated intractable disease in Japan (designated intractable disease number 223), whereas several cases with primary MPGN were registered in the registry of primary nephrotic syndrome (designated intractable disease number 222). Therefore, we combined the registry data of the primary MPGN, as shown in Fig. 1a. In the clinical records of patients with primary MPGN (designated intractable disease number 223), not primary nephrotic syndrome (designated intractable disease number 222), there are survey items about renal replacement therapy and pathological findings of a modified Japanese classification [20] (Table S1).

### Data curation

The data used in the statistical analysis were processed and refined in advance to make them suitable for analysis. If multiple patients had identical data, the data from one patient were included in the analysis, and duplicate data were excluded. Erroneous data (e.g., values that were entered although no tests were performed) were included after the likely values were assigned as alternatives. These values were determined by referring to the basic statistics of the data.

### Characteristics and management of primary MPGN

Demographics (age at onset and sex), chronic kidney disease classification based on glomerular filtration rate and albuminuria, and management of primary MPGN in this registry were determined. The use and dosage (highest dose in the past 6 months; present use only) of present and previously used steroids and immunosuppressive drugs were also collected. ‘Steroid-resistant’ was defined as never achieving complete remission despite immunosuppressive treatment, including steroid therapy, according to the definition outlined in the Japanese guidelines for nephrotic syndrome [21]. After removing patients with multiple diagnoses and missing data, we analyzed the following two sets:

1. Comparison between nephrotic and non-nephrotic patients with primary MPGN. Nephrotic syndrome was diagnosed based on massive proteinuria (≥ 3.5 g/day) and hypoalbuminemia (serum albumin ≤ 3.0 g/dL) [19].
2. Patients with MPGN were divided into three age groups: adolescent and young adults (≤ 39 years), middle adults (40–64 years), and older adults (≥ 65 years). Clinical parameters were compared among age groups.

### Statistical analysis

Quantitative variables are expressed as mean (standard deviation) for normally distributed data or median (interquartile range [IQR]). Qualitative variables are presented as frequencies (percentages). Differences between groups were compared using chi-square tests for categorical variables and nonparametric Kruskal–Wallis tests for continuous variables. All data were analyzed using IBM SPSS (version 26.0; SPSS, Chicago, IL, USA), and P < 0.05 indicated a significant difference.

## Results

In the nationwide registry of clinical personal records from 2015 to 2018, 156 and 114 cases of primary MPGN were enrolled as primary nephrotic syndrome (designated intractable disease number 222) and primary MPGN (designated intractable disease number 223), respectively. We merged and analyzed these cases (Fig. 1). After removing patients with missing data, 258 patients with primary MPGN were included in this analysis (Fig. 1a). Of 114 cases of primary MPGN (designated intractable disease number 223), 4 patients (5.3%) underwent hemodialysis for ESKD.

### Distribution and sex differences of primary MPGN based on age at onset in the national registry of clinical personal records

Regarding the distribution of age at onset, primary MPGN was present in all ages with a bimodal pattern, showing an early peak at 5–19 years and a later peak at 45–59 years (Fig. 1b). There was a female predominance in young and middle-aged individuals, whereas a male predominance was observed in older adults (Fig. 1b).

### Comparison between nephrotic and non-nephrotic patients with primary MPGN

**Fig. 2:**
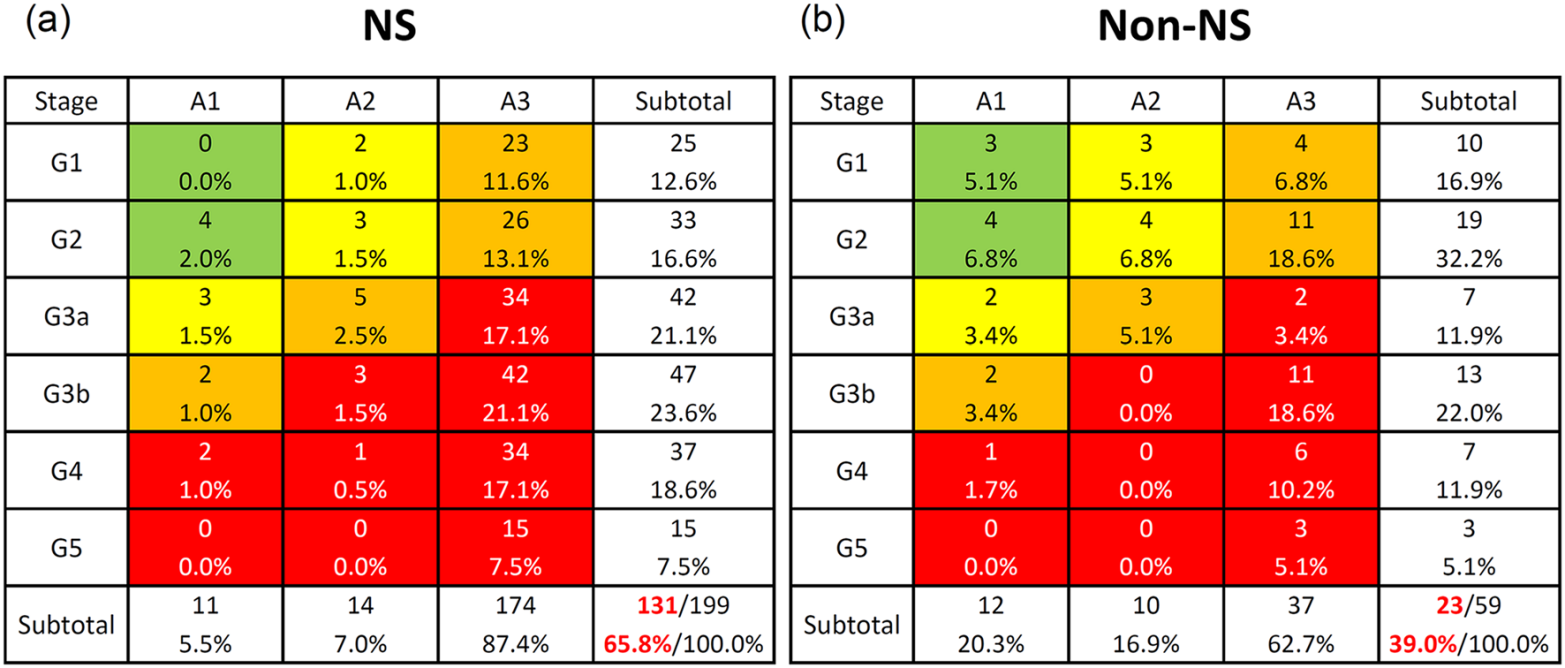
Risk classification of chronic kidney disease of nephrotic (**a**) and non-nephrotic (**b**) patients with primary membranoproliferative glomerulonephritis. Green: low risk (if no other markers of kidney disease, no chronic kidney disease); yellow: moderately increased risk; Orange: high risk; Red, very high risk

Of 258 patients with primary MPGN, 199 with nephrotic syndrome and 59 with non-nephrotic syndrome were included in this analysis (Fig. 1, Table 1). The median (IQR) age at enrollment was higher for nephrotic syndrome than for non-nephrotic syndrome (52 [36–67] vs. 47 [23–59] years, respectively; P = 0.007). The median (IQR) age at onset was also higher for nephrotic syndrome than for non-nephrotic syndrome (45 [24–63] vs. 35 [14–53] years, respectively; P = 0.010). Both the G and A stages of the risk classification of chronic kidney disease were more severe in patients with nephrotic syndrome than in those with non-nephrotic syndrome (Table 1). According to the CKD risk classification, very high-risk (red zone) patients accounted for 65.8% of patients with nephrotic syndrome (Fig. 2a) and 39.0% of those with non-nephrotic syndrome (Fig. 2b).

**Table 1:** Demographics of nephrotic and non-nephrotic syndrome in the national registry of clinical personal records of primary membranoproliferative glomerulonephritis

	All	NS	Non-NS	P value
N	258	199	59	
Age (years old)	51 (34, 65)	52 (36, 67)	47 (23, 59)	0.007
Age of onset (years old)	45 (21, 59)	45 (24, 63)	35 (14, 53)	0.010
Male, n (%)	134 (51.9%)	101 (50.8%)	33 (55.9%)	0.484
CKD G stage				
G1	35 (13.6%)	25 (12.6%)	10 (16.9%)	0.080
G2	52 (20.2%)	33 (16.6%)	19 (32.2%)	
G3a	49 (19.0%)	42 (21.1%)	7 (11.9%)	
G3b	60 (23.3%)	47 (23.6%)	13 (22.0%)	
G4	44 (17.1%)	37 (18.6%)	7 (11.9%)	
G5	18 (7.0%)	15 (7.5%)	3 (5.1%)	
CKD A stage				
A1	23 (8.9%)	11 (5.5%)	12 (20.3%)	< 0.001
A2	24 (9.3%)	14 (7.0%)	10 (16.9%)	
A3	211 (81.8%)	174 (87.4%)	37 (62.7%)	
Present treatment				
Oral prednisolone	182 (70.5%)	147 (73.9%)	35 (59.3%)	0.032
IV methylprednisolone	16 (6.2%)	16 (12.8%)	0 (0.0%)	0.064
Cyclosporine	63 (24.4%)	51 (25.6%)	12 (20.3%)	0.407
Tacrolimus	8 (3.1%)	7 (3.5%)	1 (1.7%)	0.479
Cyclophosphamide	5 (1.9%)	4 (2.0%)	1 (1.7%)	0.878
Mizoribine	38 (14.7%)	27 (13.6%)	11 (18.6%)	0.335
Mycophenolate mofetil	3 (1.2%)	3 (1.5%)	0 (0.0%)	0.344
Rituximab	10 (3.9%)	9 (4.5%)	1 (1.7%)	0.324
Dosage of present treatment				
Oral prednisolone	15.5 ± 15.1	16.4 ± 15.3	11.7 ± 14.3	0.055
IV methylprednisolone	656.3 ± 239.4	656.3 ± 239.4	–	–
Cyclosporine	89.9 ± 52.2	94.7 ± 41.4	69.6 ± 23.6	0.037
Tacrolimus	2.9 ± 0.4	2.8 ± 0.4	3.0	1.000
Cyclophosphamide	47.6 ± 33.4	47.0 ± 38.5	50.0	1.000
Mizoribine	128.3 ± 31.9	129.6 ± 28.6	125.0 ± 40.3	0.949
Mycophenolate mofetil	916.7 ± 520.4	916.7 ± 520.4	–	–
Rituximab	550.0 ± 497.0	550.0 ± 497.0	–	–
Steroid-resistant NS	152 (58.9%)	123 (62.1%)	29 (49.2%)	0.083

Data are expressed as the mean ± SD, median (interquartile range), or number (percentage). The P value was derived from the Mann–Whitney U test for NS vs. non-NS

*CKD* chronic kidney disease, *NS* nephrotic syndrome

The use of oral prednisolone was significantly higher in patients with nephrotic syndrome (73.9%) than in those with non-nephrotic syndrome (59.3%; P = 0.032) (Table 1). The mean dose of oral prednisolone in patients with nephrotic syndrome (16.4 ± 15.3 mg/day) tended to be higher than that in those with non-nephrotic syndrome (11.7 ± 14.3 mg/day; P = 0.055). The use of immunosuppressive drugs except for prednisolone was higher in patients with nephrotic syndrome than in those with non-nephrotic syndrome, but the difference was not significant (Table 1). In contrast, the mean dosage of cyclosporine was significantly higher in patients with nephrotic syndrome (94.7 ± 41.4 mg/day) than in those with non-nephrotic syndrome (69.6 ± 23.6 mg/day; P = 0.037). As for steroid responses, the steroid-resistant nephrotic syndrome was higher in patients with nephrotic syndrome than in those with non-nephrotic syndrome (62.1% vs. 49.2%), but the difference was not significant (Table 1).

### Comparison among three age groups in patients with primary MPGN

**Fig. 3:**
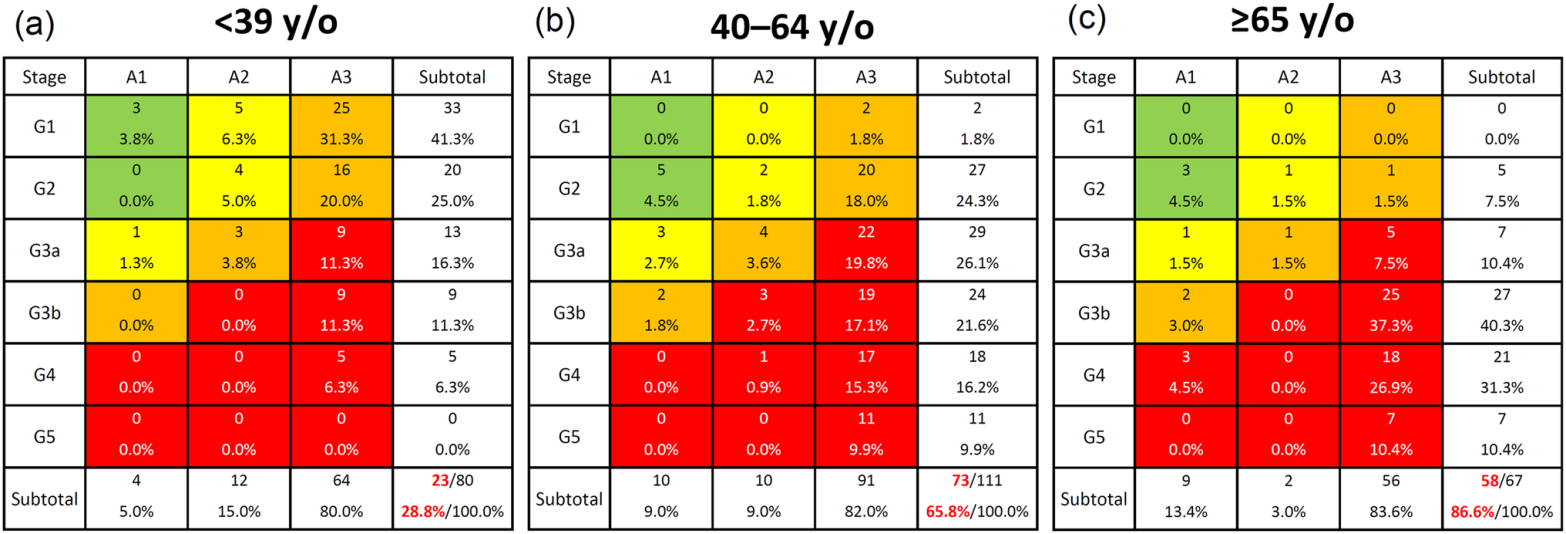
Risk classification of chronic kidney disease by three age groups in patients with primary membranoproliferative glomerulonephritis: adolescent and young adult group (≤ 39 years) (**a**), middle adult group (40–64 years) (**b**), and older adult group (≥ 65 years) (**c**). Green: low risk (if no other markers of kidney disease, no chronic kidney disease); yellow: moderately increased risk; orange: high risk; red, very high risk

Patients were divided into three age groups: adolescent and young adult (≤ 39 years), middle adult (40–64 years), and older adult (≥ 65 years) (Table 2). Females were dominant in the adolescent, young adult, and middle adult groups, whereas males were dominant in the older adult group. The frequency of nephrotic syndrome was significantly higher in the older adult group (91.0%) than in the adolescent and young adult (72.5%, P < 0.05) and middle adult (72.1%, P < 0.05) groups (Table 2). According to the CKD risk classification, very high-risk (red zone) patients accounted for 28.8% of adolescents and young adults, 65.8% of middle-aged adults, and 86.6% of older patients (Fig. 3). The use of oral prednisolone, cyclosporine, and mizoribine was significantly higher in the adolescent and young adult groups than in the middle-aged group, whereas the mean dosage of oral prednisolone and mizoribine showed no differences among the three age groups. The mean dosage of cyclosporine was significantly higher in the middle adult group than in the adolescent and young adult group (107.0 ± 46.6 vs. 75.8 ± 35.0 mg/day, P < 0.05). As for steroid responses, steroid-resistant patients tended to be higher in the adolescent and young adult group than in the middle-aged and older adult groups (70.0%, 53.7%, and 54.1%, respectively), but the difference was not significant.

**Table 2:** Demographics of cases with primary membranoproliferative glomerulonephritis according to age

	< 39 y/o	40–64 y/o	≥ 65 y/o	P value
N	80	111	67	
Age of enrollment (years old)	28 (21, 34)	52 (47, 57)	70 (67, 76)	< 0.001^a,b,c^
Age of onset (years old)	16 (12, 24)	46 (37, 54)	68 (64, 71)	< 0.001^a,b,c^
Male	39 (48.8%)	54 (48.6%)	41 (61.2%)	0.213
Nephrotic syndrome	58 (72.5%)	80 (72.1%)	61 (91.0%)	0.007^b,c^
Present treatment				
Oral prednisolone	64 (80.0%)	70 (63.1%)	48 (71.6%)	0.040^a^
IV methylprednisolone	5 (6.3%)	2 (1.8%)	9 (13.4%)	0.028^c^
Cyclosporine	29 (36.3%)	23 (20.7%)	11 (16.4%)	0.010^a,b^
Tacrolimus	3 (3.8%)	5 (4.5%)	0 (0.0%)	0.226
Cyclophosphamide	1 (1.3%)	2 (1.8%)	2 (3.0%)	0.743
Mizoribine	22 (27.5%)	9 (8.1%)	7 (10.4%)	0.001^a,b^
Mycophenolate mofetil	3 (3.8%)	0 (0.0%)	0 (0.0%)	0.035
Rituximab	3 (3.8%)	3 (2.7%)	4 (6.0%)	0.549
Dosage of present treatment				
Oral prednisolone (mg/day)	15.4 ± 16.4	14.2 ± 14.0	17.6 ± 15.1	0.495
IV methylprednisolone (mg/day)	800.0 ± 273.9	500.0 ± 0.0	611.1 ± 220.5	0.226
Cyclosporine (mg/day)	75.8 ± 35.0	107.0 ± 46.6	91.4 ± 19.1	0.043^a^
Tacrolimus (mg/day)	2.7 ± 0.6	3.0 ± 0.0	–	0.248
Cyclophosphamide (mg/day)	100.0	50.0 ± 20.4	19.0 ± 8.5	0.150
Mizoribine (mg/day)	125.0 ± 37.0	147.2 ± 8.3	114.3 ± 24.4	0.054
Mycophenolate mofetil (mg/day)	916.7 ± 520.4	–	–	–
Rituximab (mg/month)	500 ± 0.0	366.7 ± 230.9	733.3 ± 680.7	0.487
Steroid-resistant	56 (70.0%)	36 (53.7%)	60 (54.1%)	0.053

Data are expressed as the mean ± SD, median (interquartile range), or number (percentage)

*NS* nephrotic syndrome

^a^P < 0.05, < 39 y/o vs. 40–64 y/o. ^b^P < 0.05, < 39 y/o vs. ≥ 65 y/o. ^c^P < 0.05, 40–64 y/o vs. ≥ 65 y/o. Kruskal–Wallis tests with Bonferroni-corrected P-values

### Pathological findings in patients with primary MPGN

Of the 114 cases of primary MPGN (designated intractable disease number 223), pathology findings were entered in 88 cases. The most common finding was moderate diffuse and global double contours of glomerular basement membrane (GBM) (26.1%), followed by diffuse and global lobular appearance with double contours of GBM (20.5%) (Table S2). In a comparison of pathological findings between nephrotic and non-nephrotic patients with primary MPGN, the degree of arteriosclerosis was significantly higher in nephrotic patients than in non-nephrotic patients; age was also higher in nephrotic patients than in non-nephrotic patients (Table S2).

## Discussion

This study is the first to describe the clinical features and real-world management of patients with primary MPGN throughout Japan, including both the use and mean dosage of immunosuppressants, according to a national database with more than 250 patients.

The incidence of MPGN has been reported to range from 1.4 to 9.3 pmp/year [3], with a few exceptions. Moreover, the incidence has decreased over time (from about seven cases pmp in the 1970s to about two cases pmp in the 1990–2000 period) [22, 23]. The decline in primary MPGN from the 1970s to the 2000s has also been reported in Japan [8]. In a comprehensive epidemiological study in Australia in 2001 [24], MPGN lesions were observed in approximately 2–3 pmp in children (aged 5–14 years) and in approximately 1–10 pmp in adults (aged 14–75+ years), and the peak incidence of MPGN was observed in patients aged 55–74 years. In the present study, using the national registry of clinical personal records, primary MPGN was present in all ages with a bimodal pattern, showing an early peak at 5–19 years and a later peak at 45–59 years, suggesting that the age distribution in Japan is similar to that worldwide, although no data on the incidence pmp was available in Japan. A European study of patients with MPGN reported that those with higher age and more prevalent glomerular sclerosis at diagnosis had inferior renal outcomes [25]. We have also reported that higher age (per 10 years) was an independent predictor for progression to ESKD in Japanese patients with primary MPGN [12]. However, prognosis is influenced by various factors, including histopathological findings, presence of comorbidities (e.g., hypertension or diabetes), and treatment response. Therefore, further large and longitudinal cohort are needed to obtain more accurate prognosis of young-onset and post-middle-age-onset MPGN.

Using the J-RBR, we have previously reported the clinicopathological findings of primary MPGN, showing that middle and older adults with primary MPGN had a higher prevalence of nephrotic syndrome, clinical hypertension, heavy proteinuria, and hypoalbuminemia at the time of biopsy than children with primary MPGN [11]. In the present study, the ratios of nephrotic syndrome among the three age groups (62.9 vs. 16.7 vs. 48.8%) were significantly higher, whereas those of chronic nephritic syndrome (29.4 vs. 76.7 vs. 46.5%) were significantly lower, in older adults than in children and middle adults, respectively. Similar to the J-RBR, the median age at onset was higher for nephrotic than for non-nephrotic syndrome (45 vs. 35 years, respectively; P = 0.010, Table 1) in the present study. Furthermore, we showed that the use of oral prednisolone was significantly higher in patients with nephrotic syndrome (73.9%) than in those with non-nephrotic syndrome (59.3%), whereas the mean dose of oral prednisolone was not significantly different between the two groups. Several previous reports in Japan also reported the use of oral prednisolone at 70–80% [8, 12], but they did not show that according to nephrotic or non-nephrotic syndrome.

Regarding the comparison between the three age groups, adolescent and young adult (≤ 39 years) patients with primary MPGN received a higher prescription rate of oral prednisolone, cyclosporine, and mizoribine than middle adult (40–64 years) patients, suggesting that steroid-resistant nephrotic syndrome is more common in adolescent and young adult patients. In contrast, the mean dosages of oral prednisolone and mizoribine showed no differences among the three age groups, because nephrotic syndrome was more common in older adults; therefore, immunosuppressive treatment was required even in older adults with primary MPGN.

This study has several limitations. First, the database does not contain data on complications (e.g., hypertension and diabetes), genetic testing, non-pharmacological therapy, and patient outcomes. Furthermore, the database does not contain data about urine protein levels, estimated glomerular filtration rate, serum albumin, and complement levels. Second, we demonstrated the characteristics of primary MPGN throughout Japan using descriptive analysis. However, temporal trend analysis would provide more clinically relevant findings. Further studies using temporal trend analysis are needed to reveal longitudinal demographic changes and the rate of guideline-directed medical treatment. Third, the pathological findings were only available in about one-third of all cases. Fourth, we could not distinguish between immune complex-mediated MPGN and C3 nephropathy [1] because of the lack of immunostaining findings in this study. Despite these limitations, we believe that the outcomes of this study will help provide an insightful overview of the clinical characteristics and features of patients with primary MPGN throughout Japan, in addition to developing the optimal management for these patients.

In conclusion, by analyzing a nationwide database of more than 250 patients with primary MPGN in Japan, this study revealed their clinical characteristics and provided critical insights for developing optimal management strategies for primary MPGN, which is a rare cause of nephrotic syndrome and ESKD. Further investigations are needed to improve the therapeutic strategies against primary MPGN in Japan.

### Supplementary Information

Below is the link to the electronic supplementary material.

Supplementary file1 (DOCX 34 KB)

## Data Availability

The datasets generated and analyzed during the current study are not publicly available because the consent obtained from the participants does not cover the unlimited public sharing of the data.

## References

[1] Sethi S, Fervenza FC (2012). Membranoproliferative glomerulonephritis–a new look at an old entity. N Engl J Med.

[2] Kidney Disease: Improving Global Outcomes Glomerular Diseases Work G (2021). KDIGO 2021 Clinical Practice Guideline for the Management of Glomerular Diseases. Kidney Int.

[3] McGrogan A, Franssen CF, de Vries CS (2011). The incidence of primary glomerulonephritis worldwide: a systematic review of the literature. Nephrol Dial Transplant.

[4] Sugiyama H, Yokoyama H, Sato H, Saito T, Kohda Y, Nishi S (2011). Japan Renal Biopsy Registry: the first nationwide, web-based, and prospective registry system of renal biopsies in Japan. Clin Exp Nephrol.

[5] Sugiyama H, Yokoyama H, Sato H, Saito T, Kohda Y, Nishi S (2013). Japan Renal Biopsy Registry and Japan Kidney Disease Registry: committee report for 2009 and 2010. Clin Exp Nephrol.

[6] Yamamoto R, Imai E, Maruyama S, Yokoyama H, Sugiyama H, Nitta K (2020). Incidence of remission and relapse of proteinuria, end-stage kidney disease, mortality, and major outcomes in primary nephrotic syndrome: the Japan Nephrotic Syndrome Cohort Study (JNSCS). Clin Exp Nephrol.

[7] Iitaka K, Ishidate T, Hojo M, Kuwao S, Kasai N, Sakai T (1995). Idiopathic membranoproliferative glomerulonephritis in Japanese children. Pediatr Nephrol.

[8] Kawamura T, Usui J, Kaseda K, Takada K, Ebihara I, Ishizu T (2013). Primary membranoproliferative glomerulonephritis on the decline: decreased rate from the 1970s to the 2000s in Japan. Clin Exp Nephrol.

[9] Okuda Y, Ishikura K, Hamada R, Harada R, Sakai T, Hamasaki Y (2015). Membranoproliferative glomerulonephritis and C3 glomerulonephritis: frequency, clinical features, and outcome in children. Nephrology (Carlton).

[10] Kawasaki Y, Kanno S, Ono A, Suzuki Y, Ohara S, Sato M (2016). Differences in clinical findings, pathology, and outcomes between C3 glomerulonephritis and membranoproliferative glomerulonephritis. Pediatr Nephrol.

[11] Nakagawa N, Hasebe N, Hattori M, Nagata M, Yokoyama H, Sato H (2018). Clinical features and pathogenesis of membranoproliferative glomerulonephritis: a nationwide analysis of the Japan renal biopsy registry from 2007 to 2015. Clin Exp Nephrol.

[12] Nakagawa N, Mizuno M, Kato S, Maruyama S, Sato H, Nakaya I (2021). Demographic, clinical characteristics and treatment outcomes of immune-complex membranoproliferative glomerulonephritis and C3 glomerulonephritis in Japan: a retrospective analysis of data from the Japan Renal Biopsy Registry. PLoS ONE.

[13] Nakagawa N, Sofue T, Kanda E, Nagasu H, Matsushita K, Nangaku M (2020). J-CKD-DB: a nationwide multicentre electronic health record-based chronic kidney disease database in Japan. Sci Rep.

[14] Sofue T, Nakagawa N, Kanda E, Nagasu H, Matsushita K, Nangaku M (2020). Prevalence of anemia in patients with chronic kidney disease in Japan: a nationwide, cross-sectional cohort study using data from the Japan Chronic Kidney Disease Database (J-CKD-DB). PLoS ONE.

[15] Nagasu H, Yano Y, Kanegae H, Heerspink HJL, Nangaku M, Hirakawa Y (2021). Kidney outcomes associated with SGLT2 inhibitors versus other glucose-lowering drugs in real-world clinical practice: the japan chronic kidney disease database. Diabetes Care.

[16] Enzan N, Matsushima S, Ide T, Kaku H, Tohyama T, Funakoshi K (2021). Clinical characteristics and contemporary management of patients with cardiomyopathies in Japan—Report from a national registry of clinical personal records. Circ Rep.

[17] Hayashida M, Kinjo T, Wada Y, Kitaguchi Y, Hanaoka M (2022). Hierarchical cluster analysis based on disease-associated manifestations of patients with lymphangioleiomyomatosis: an analysis of the national database of designated intractable diseases of Japan. Respir Investig.

[18] Kimura T, Ikeuchi H, Yoshino M, Sakate R, Maruyama S, Narita I (2023). Profiling of kidney involvement in systemic lupus erythematosus by deep learning using the National Database of Designated Incurable Diseases of Japan. Clin Exp Nephrol.

[19] Wada T, Ishimoto T, Nakaya I, Kawaguchi T, Sofue T, Shimizu S (2021). A digest of the evidence-based clinical practice guideline for nephrotic syndrome 2020. Clin Exp Nephrol.

[20] Sakaguchi H, Hazikano H, Hasegawa O, Ito H (1984). Histological subtype of mesangiocapillary glomerulonephritis (MPGN type 1). Nihon Jinzo Gakkai Shi.

[21] Nishi S, Ubara Y, Utsunomiya Y, Okada K, Obata Y, Kai H (2016). Evidence-based clinical practice guidelines for nephrotic syndrome 2014. Clin Exp Nephrol.

[22] Braden GL, Mulhern JG, O'Shea MH, Nash SV, Ucci AA, Germain MJ (2000). Changing incidence of glomerular diseases in adults. Am J Kidney Dis.

[23] Zhou FD, Zhao MH, Zou WZ, Liu G, Wang H (2009). The changing spectrum of primary glomerular diseases within 15 years: a survey of 3331 patients in a single Chinese centre. Nephrol Dial Transplant.

[24] Briganti EM, Dowling J, Finlay M, Hill PA, Jones CL, Kincaid-Smith PS (2001). The incidence of biopsy-proven glomerulonephritis in Australia. Nephrol Dial Transplant.

[25] Garam N, Prohaszka Z, Szilagyi A, Aigner C, Schmidt A, Gaggl M (2020). Validation of distinct pathogenic patterns in a cohort of membranoproliferative glomerulonephritis patients by cluster analysis. Clin Kidney J.

