# Supplementary material for: Demographics and treatment of patients with primary membranoproliferative glomerulonephritis in Japan using a national registry of clinical personal records
Source: Clin Exp Nephrol. 2023 Jul 29;27(11):928–35. doi: 10.1007/s10157-023-02387-1 (PMC10581954; doi:10.1007/s10157-023-02387-1)
Supplement: Supplementary file 1 — Supplementary file1 (DOCX 34 KB) [file 10157_2023_2387_MOESM1_ESM.docx]

**Demographics and Treatment of Patients with Primary Membranoproliferative Glomerulonephritis in Japan Using a National Registry of Clinical Personal Records**

Naoki Nakagawa^1^*, Tomonori Kimura^2,3^, Ryuichi Sakate^2^, Yoshitaka Isaka^4^, and Ichiei Narita^5^

^1^Division of Cardiology, Nephrology, Pulmonology and Neurology, Department of Internal Medicine, Asahikawa Medical University, Asahikawa, Japan

^2^Reverse Translational Research Project, Center for Rare Disease Research, National Institutes of Biomedical Innovation, Health and Nutrition (NIBIOHN), Ibaraki, Japan

^3^Laboratory of Rare Disease Resource Library, Center for Rare Disease Research, National Institutes of Biomedical Innovation, Health and Nutrition (NIBIOHN), Ibaraki, Japan

^4^Department of Nephrology, Osaka University Graduate School of Medicine, Suita, Japan.

^5^Division of Clinical Nephrology and Rheumatology, Kidney Research Center, Niigata University Graduate School of Medical and Dental Sciences, Niigata, Japan

*Corresponding author: Naoki Nakagawa, MD, PhD, Division of Cardiology, Nephrology, Pulmonology and Neurology, Department of Internal Medicine, Asahikawa Medical University, 2-1-1-1 Midorigaoka-higashi, Asahikawa, Japan, Phone: +81-166-68-2442, Fax: +81-166-68-2449, E-mail: [naka-nao@asahikawa-med.ac.jp](mailto:naka-nao@asahikawa-med.ac.jp) (NN)

Table S1. Survey items of pathological findings of primary membranoproliferative glomerulonephritis in the clinical personal records.

| 1. Glomerular injury pattern of membranoproliferative glomerulonephritis |
| --- |
| 1. Mesangial proliferation |
| □ 1a. slight |
| □ 1b. moderate |
| 2. Chronic and focal |
| □ focal, segmental or global mesangial proliferation and double contours of the GBM |
| 3. Acute and focal |
| □ focal, segmental or global mesangial and endocapillary proliferation with inflammatory cells (neutrophil) without double contours of GBM |
| 4. Chronic and diffuse |
| □ 4a. slight segmental double contours of GBM |
| □ 4b.non-lobular, diffuse and global double contours of GBM |
| □ 4c.moderate diffuse and global double contours of GBM |
| 5. Acute and diffuse |
| □ 5a. endocapillary proliferation with inflammatory cells (neutrophil) |
| □ 5b. moderate endocapillary proliferation with subendothelial deposits |
| □ 5c. marked endocapillary proliferation with subendothelial deposits |
| 6. Lobular |
| □ diffuse and global lobular appearance with double contours of GBM with nodular mesangial sclerosis in the moderately enlarged glomeruli |
| 7. End stage |
| □ diffuse and global glomerulosclerosis |
| 1. Additional findings |
| 1. Glomerular lesion |
| A.　Global sclerotic lesion ％，Segmental sclerotic lesion ％  B.　Crescentic lesion　　　　 ％  C.　Degree of infiltration of white blood cells 1. － 2. ± 3. ＋ 4. ＋＋  D.　Degree of infiltration of foam cells　　　 1. － 2. ± 3. ＋ 4. ＋＋  E.　Degree of subendothelial deposits　　 1. － 2. ± 3. ＋ 4. ＋＋ |
| 1. Tubulointerstitial lesion |
| A.　Tubulointerstitial change in the cortex（％）　　　　　　　％  B.　Degree of foam cellularization of tubular epithelium 1.－ 2.± 3.＋ 4.＋＋ |
| 1. Vascular lesion |
| Degree of arteriosclerosis　　 1. － 2. ± 3. ＋ 4. ＋＋ |

GBM, glomerular basement membrane.

Table S2. Pathological findings of primary membranoproliferative glomerulonephritis

|  | All | NS | Non-NS | P value |
| --- | --- | --- | --- | --- |
| N | 88 | 62 | 26 |  |
| Age (y) | 51 (28, 63) | 54 (38, 65) | 44 (21, 55) | 0.011 |
| Glomerular injury pattern |  |  |  |  |
| 1. Mesangial proliferation |  |  |  |  |
| 1a. slight | 3 (3.4%) | 2 (3.2%) | 1 (3.8%) | 0.665 |
| 1b. moderate | 6 (6.8%) | 5 (8.1%) | 1 (3.8%) | 0.665 |
| 2. Chronic and focal | 12 (13.6%) | 8 (12.9%) | 4 (15.4%) | 0.917 |
| 3. Acute and focal | 4 (4.5%) | 1 (1.6%) | 3 (11.5%) | 0.076 |
| 4. Chronic and diffuse |  |  |  |  |
| 4a. slight | 10 (11.4%) | 4 (6.5%) | 6 (23.1%) | 0.417 |
| 4b.non-lobular | 7 (8.0%) | 4 (6.5%) | 3 (11.5%) | 0.417 |
| 4c. moderate | 23 (26.1%) | 19 (30.6%) | 4 (15.4%) | 0.186 |
| 5. Acute and diffuse |  |  |  |  |
| 5a. endocapillary | 1 (1.1%) | 1 (1.6%) | 0 (0.0%) | 0.705 |
| 5b. moderate | 4 (4.5%) | 3 (4.8%) | 1 (3.8%) | 0.661 |
| 5c. marked | 0 (0.0%) | 0 (0.0%) | 0 (0.0%) | - |
| 6. Lobular | 18 (20.5%) | 15 (24.2%) | 3 (11.5%) | 0.250 |
| 7. End stage | 0 (0.0%) | 0 (0.0%) | 0 (0.0%) | - |
| Additional findings |  |  |  |  |
| Glomerular lesion |  |  |  |  |
| Global sclerotic lesion | 12.5 ± 13.8% | 13.2 ± 14.7% | 10.2 ± 14.3% | 0.381 |
| Segmental sclerotic lesion | 4.9 ± 14.7% | 6.4 ± 15.8% | 1.8 ± 5.2% | 0.172 |
| Crescentic lesion | 5.7 ± 12.7% | 4.8 ± 9.2% | 9.6 ± 21.0% | 0.975 |
| Infiltration of white blood cells* | 2.2 ± 1.0 | 2.3 ± 0.9 | 2.2 ± 1.0 | 0.569 |
| Infiltration of foam cells* | 1.9 ± 0.9 | 1.8 ± 0.8 | 2.1 ± 1.1 | 0.186 |
| Subendothelial deposits* | 2.8 ± 1.0 | 3.0 ± 0.9 | 2.9 ± 1.0 | 0.683 |
| Tubulointerstitial lesion |  |  |  |  |
| Tubulointerstitial change in the cortex | 23.6 ± 19.5% | 23.2 ± 15.5% | 24.0 ± 28.9% | 0.195 |
| Foam cellularization of tubular epithelium* | 1.8 ± 0.9 | 1.8 ± 0.8 | 2.0 ± 1.1 | 0.482 |
| Vascular lesion |  |  |  |  |
| Arteriosclerosis* | 2.1 ± 1.0 | 2.3 ± 1.0 | 1.6 ± 0.8 | 0.020 |

Data are expressed as the mean ± SD, median (interquartile range), or number (percentage). The P-value was derived from the Mann-Whitney U test for NS vs. non-NS. NS, nephrotic syndrome.

*Score of degree: 1-4.
